# Self-programmed enzyme phase separation and multiphase coacervate droplet organization†

**DOI:** 10.1039/d0sc06418a

**Published:** 2021-01-25

**Authors:** Hedi Karoui, Marianne J. Seck, Nicolas Martin

**Affiliations:** Univ. Bordeaux, CNRS, Centre de Recherche Paul Pascal, UMR5031 115 Avenue du Dr Schweitzer 33600 Pessac France nicolas.martin@crpp.cnrs.fr

## Abstract

Membraneless organelles are phase-separated droplets that are dynamically assembled and dissolved in response to biochemical reactions in cells. Complex coacervate droplets produced by associative liquid–liquid phase separation offer a promising approach to mimic such dynamic compartmentalization. Here, we present a model for membraneless organelles based on enzyme/polyelectrolyte complex coacervates able to induce their own condensation and dissolution. We show that glucose oxidase forms coacervate droplets with a cationic polysaccharide on a narrow pH range, so that enzyme-driven monotonic pH changes regulate the emergence, growth, decay and dissolution of the droplets depending on the substrate concentration. Significantly, we demonstrate that time-programmed coacervate assembly and dissolution can be achieved in a single-enzyme system. We further exploit this self-driven enzyme phase separation to produce multiphase droplets *via* dynamic polyion self-sorting in the presence of a secondary coacervate phase. Taken together, our results open perspectives for the realization of programmable synthetic membraneless organelles based on self-regulated enzyme/polyelectrolyte complex coacervation.

## Abstract 2

Self-programmed enzyme phase separation is exploited to assemble dynamic multiphase coacervate droplets *via* spontaneous polyion self-sorting under non-equilibrium conditions.

## Graphical abstract

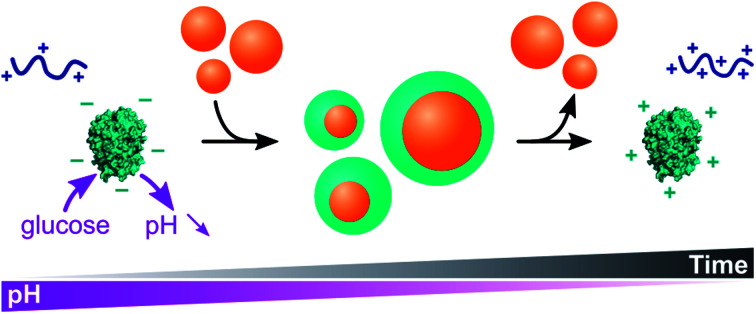

## Introduction

Liquid–liquid phase separation (LLPS) of biological polymers has emerged as a ubiquitous phenomenon in the formation of membraneless organelles in living cells.^1–3^ These biomolecular condensates participate in the organization of intracellular contents,^2^ favor dynamic molecular exchanges with their environment,^3^ and can exhibit a multi-layered structure that contributes to the spatiotemporal regulation of biochemical reactions.^4,5^ A critical feature of these biological assemblies is their ability to reversibly form and dissolve in response to biochemical reactions, such as post-translational modifications or DNA transcription,^3,6–9^ which enables spatiotemporal control over the compartmentalization of biomolecules and reactions in the cytoplasm.

Drawing inspiration from nature, *in vitro* LLPS steps into the spotlight as a viable strategy for the bottom-up construction of synthetic membraneless organelles.^10–15^ Complex coacervate micro-droplets produced by associative LLPS between oppositely charged polyions recapitulate most of the features of biomolecular condensates: they exhibit selective solute uptake,^16–19^ accelerate biochemical reactions,^20–23^ and readily form or dissolve in response to physicochemical stimuli, including changes in pH,^22,23^ temperature,^24–26^ ionic strength,^27^ and under light irradiation.^28,29^ Yet, emulating both the spatial and temporal complexity of biomolecular condensates in synthetic coacervates remains challenging.

Recent directions have been geared towards increasing the spatial complexity of coacervates *via* the formation of multiphase droplets under thermodynamic equilibrium conditions,^30–32^ including by programming molecular interactions.^33,34^ On the other hand, active processes such as enzyme^35–39^ and chemical reactions^40^ have started being explored to increase the complexity of the temporal dynamics of coacervate droplets. Pioneering studies have demonstrated the use of two antagonistic enzymes acting as endogenous catalytic controllers to trigger either the condensation or dissolution of coacervates.^36,37,41^ Yet, attempts to combine both an active regulation of coacervate droplets and their spatial organization into hierarchical droplets have not yet been reported.

Our strategy relies on the use of enzymes as “scaffold” macroions to assemble self-regulating complex coacervate droplets. Studies on such a protein/polyelectrolyte coacervation phenomenon have been limited so far to single-phase systems at thermodynamic equilibrium.^17,42–48^ Here, we show that an enzyme, glucose oxidase (GOx), acts as a catalytically active “scaffold” coacervate component able to self-modulate its phase separation with an oppositely charged polysaccharide, diethylaminoethyl-dextran (DEAE-dextran). Specifically, we show that GOx and DEAE-dextran form coacervate micro-droplets on a narrow pH range corresponding to conditions close to charge stoichiometry, and exploit this pH-responsive behavior to demonstrate programmed assembly and dissolution of coacervates based on GOx-driven pH decrease in the presence of glucose. Significantly, the amount of glucose fuel supplied to the system controls the amplitude of the pH decrease so that either stable or transient assembly of coacervate droplets with controllable lifetime, together with multiple cycles of transient coacervation, are achieved. We further exploit such enzyme-responsive dynamic coacervates to create non-equilibrium multiphase droplets in the presence of a secondary coacervating system, which, to the best of our knowledge, have not been reported yet. Overall, our results highlight opportunities for the realization of self-actuated enzyme/polyelectrolyte phase separation, together with enzyme-driven polyion self-sorting into multiphase complex coacervate droplets under non-equilibrium conditions, providing new approaches to the construction of programmable synthetic membraneless organelles with increased spatiotemporal complexity.

## Results and discussion

### Formation of pH-responsive enzyme-based coacervate droplets

**Fig. 1:**
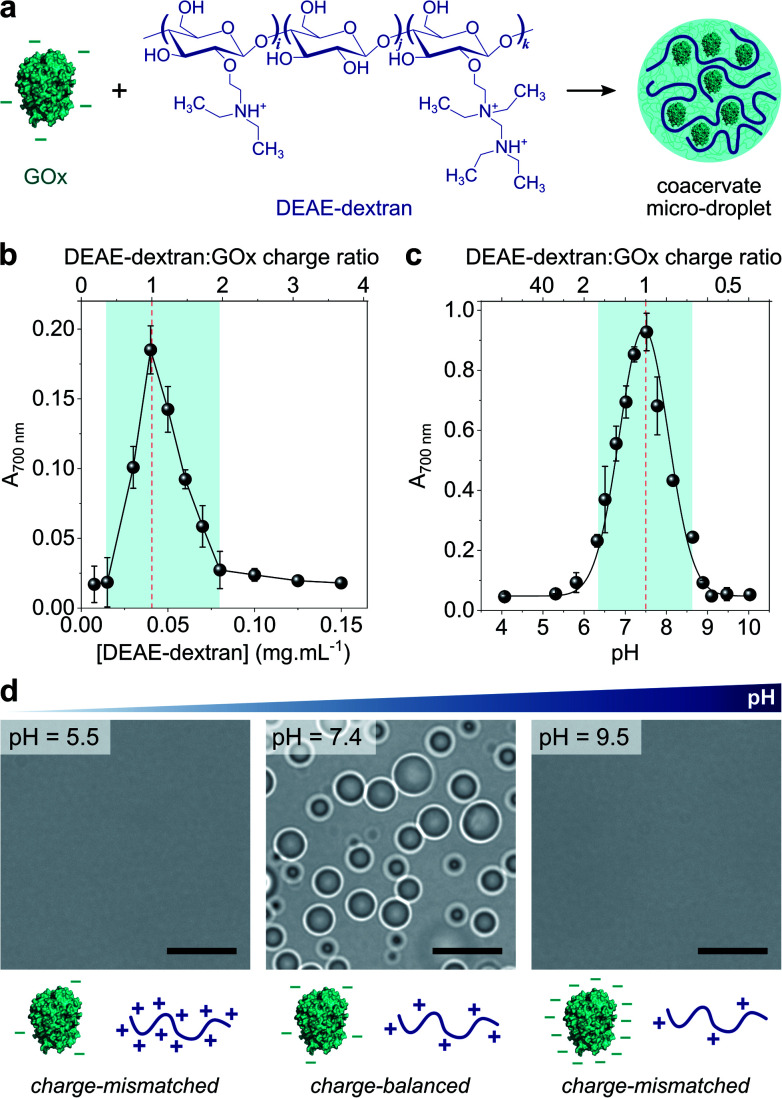
(a) Scheme of the complex coacervation process between glucose oxidase (GOx) and diethylaminoethyl (DEAE)-dextran. (b) Plot of the absorbance at 700 nm of solutions of GOx (0.25 mg mL^−1^) in the presence of varying concentrations of DEAE-dextran in phosphate buffer (2.5 mM, pH 7.4). The maximum turbidity (red dotted line) corresponds to the optimal ratio for coacervation. (c) Plot of the absorbance at 700 nm of a solution of GOx (0.25 mg mL^−1^) and DEAE-dextran (0.04 mg mL^−1^) as a function of the pH. The maximum turbidity (red dotted line) corresponds to the optimal pH for coacervation. On (b) and (c), the DEAE-dextran (positive) : GOx (negative) molar charge ratio is also reported (see ESI Note 1†). Error bars represent the standard deviation of three independent repeats. (d) Optical microscopy images of GOx/DEAE-dextran mixtures ([GOx] = 0.25 mg mL^−1^, [DEAE-dextran] = 0.04 mg mL^−1^) prepared at different pH, as indicated, and corresponding schematic representations of the charge conditions on both polyions. Scale bars, 10 μm.

Coacervate micro-droplets were first produced at equilibrium as a turbid aqueous suspension *via* liquid–liquid phase separation between DEAE-dextran and GOx at physiological pH (phosphate buffer, 2.5 mM, pH = 7.4; Fig. 1a). Turbidity measurements at varying polyion ratio (Fig. 1b), together with charge titration and calculation studies, revealed that phase separation occurred near charge neutrality (ESI Note 1 and Fig. S1†), consistent with previous studies on protein/polyelectrolyte coacervates.^17,42–48^ Optical microscopy images of the suspension produced at equimolar charge ratio (corresponding to conditions leading to maximum turbidity) confirmed the presence of polydisperse spherical micro-droplets (Fig. 1d) that fused on contact (ESI Fig. S2†), as expected for a liquid-like state. The droplets contained ∼80% of GOx molecules (ESI Fig. S3†) and readily disassembled upon increasing the ionic strength (ESI Fig. S4†), which confirmed the central role of electrostatic interactions in the phase separation process. Confocal fluorescence microscopy of coacervate droplets doped with fluorescein isothiocyanate (FITC)-labelled DEAE-dextran and rhodamine isothiocyanate (RITC)-tagged GOx further showed that the two polyions distributed homogeneously throughout the droplets (ESI Fig. S5†).

Since both polyions are weak polyelectrolytes, their net charge and charge density strongly depends on the pH. Phase behaviour of GOx/DEAE-dextran mixtures was therefore examined over a broad range of pH values at fixed protein : polymer ratio ([GOx] = 0.25 mg mL^−1^, [DEAE-dextran] = 0.04 mg mL^−1^) (Fig. 1c and d). We observed that phase separation occurred on a relatively narrow pH range (6.5 ≤ pH ≤ 8.5) corresponding to conditions close to charge neutralization between GOx (negative net charge) and DEAE-dextran (positive net charge) (ESI Fig. S1†). In comparison, coacervation was inhibited at high and low pH values due to charge mismatch between DEAE-dextran and GOx (Fig. 1c and d). These empirical observations correlated well with charge titration and calculation studies: notably, we observed that the optimal coacervation pH shifted to higher or lower values when we altered the protein : polycation ratio, an observation that could be well-predicted (ESI Note 1 and Fig. S1†). Overall, these results establish that GOx and DEAE-dextran form coacervate micro-droplets on a relatively narrow pH range and that the optimal pH for phase separation corresponds to charge neutralization conditions.

### Enzyme-driven reversible formation and dissolution of coacervate droplets

**Fig. 2:**
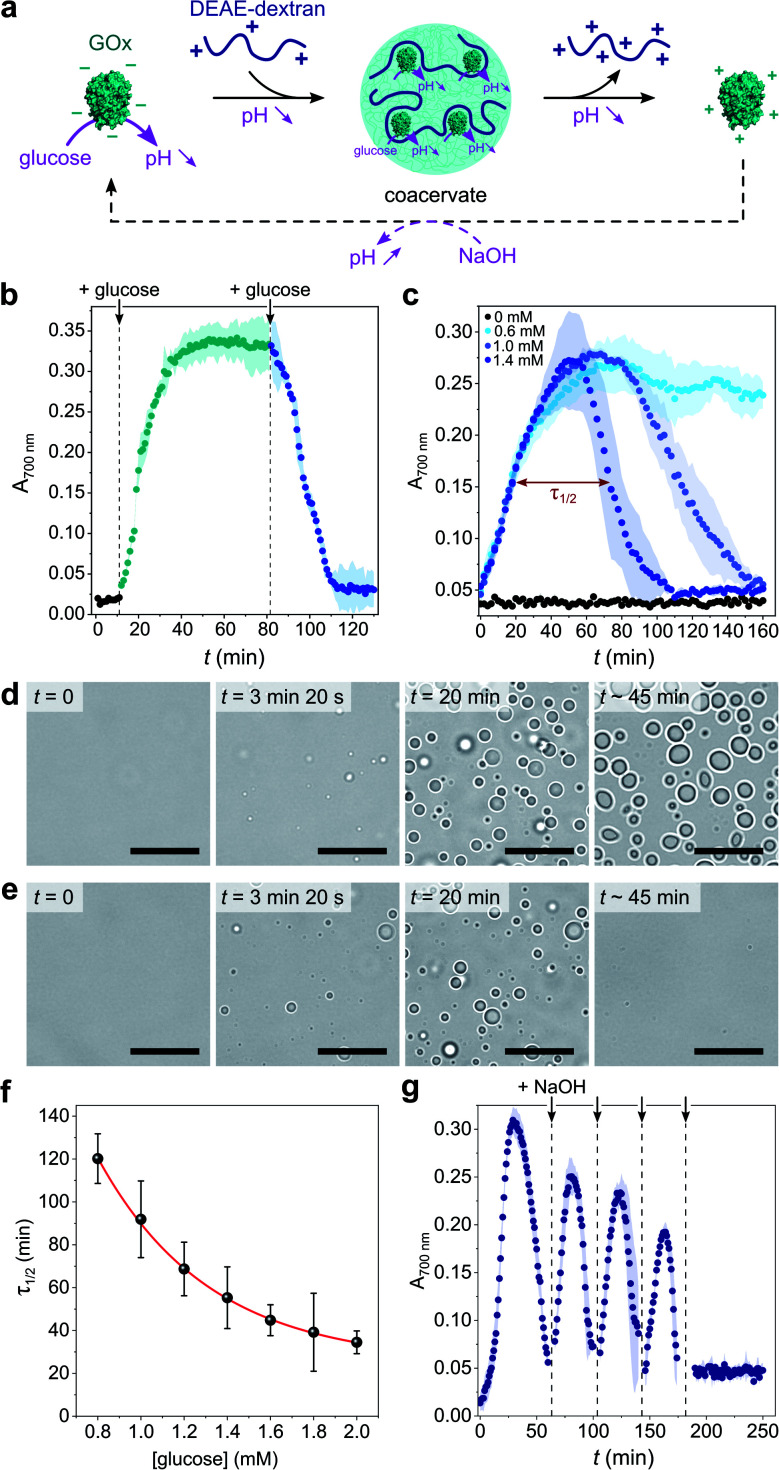
(a) Schematic representation of enzyme-mediated self-regulated complex coacervation of GOx with DEAE-dextran in the presence of glucose. GOx catalyzes the oxidation of glucose into gluconolactone that spontaneously hydrolyses into gluconic acid, producing a pH decrease that can drive coacervate formation and dissolution. (b) Time-dependent evolution of the absorbance at 700 nm of a solution of GOx (0.25 mg mL^−1^) and DEAE-dextran (0.04 mg mL^−1^) produced at pH 10.2 after the sequential addition of glucose (0.6 mM at each addition). The colored area represents error as the standard deviation of three independent repeats. (c) Time-dependent evolution of the absorbance at 700 nm of a solution of GOx (0.25 mg mL^−1^) and DEAE-dextran (0.04 mg mL^−1^) produced at pH 10.2 after the single-step addition of varying final glucose concentrations, as indicated. Above a certain glucose concentration, a bell-shape is observed, attributed to the nucleation, grow, decay and dissolution of coacervate droplets. In such conditions, *τ*_1/2_ denotes the full width at half maximum turbidity (here shown on the example of 1.4 mM glucose). The colored area represents error as the standard deviation of three independent repeats. (d and e) Optical microscopy snapshots of GOx/DEAE-dextran mixtures ([GOx] = 0.4 mg mL^−1^, [DEAE-dextran] = 0.064 mg mL^−1^) prepared at pH 10.2 at different times after addition of 0.5 mM (d) or 25 mM (e) glucose, showing the formation of stable or transient coacervate droplets, respectively. Scale bars, 20 μm. (f) Evolution of *τ*_1/2_ as defined in c as a function of the final glucose concentration. The red line represents a mono-exponential fit of the data. Error bars represent standard deviations of three independent repeats. (g) Time-dependent evolution of the absorbance at 700 nm of a solution of GOx (0.25 mg mL^−1^) and DEAE-dextran (0.04 mg mL^−1^) produced at pH 10.2 after the single-step addition of 5 mM glucose and the repeated additions of 10 mM NaOH (black arrows). The dilution factor after the last NaOH addition was ∼1.05, so the final concentrations of components did not appreciably change.

Given the above observations, we then sought to control coacervate formation in response to pH changes resulting from GOx's catalytic activity (Fig. 2a). GOx catalyzes the oxidation of glucose into gluconolactone, which spontaneously hydrolyses into gluconic acid in solution, thereby inducing a pH decrease in the absence of buffer. We first proceeded to investigate the programmed formation and disassembly of liquid droplets *via* the sequential addition of a fixed amount of glucose to mixtures of GOx (0.25 mg mL^−1^) and DEAE-dextran (0.04 mg mL^−1^) prepared at pH ∼ 10 in pure water. Addition of 0.6 mM glucose (final concentration) initiated the formation of coacervate micro-droplets, as observed by the gradual increase in the sample's turbidity, reaching a plateau after ∼40 min (Fig. 2b). This increase in turbidity was associated with a decrease in pH that stabilized around 7.4 (ESI Fig. S6†), a value close to the optimal coacervation pH for this protein : polycation ratio. In comparison, addition of another 0.6 mM glucose to this turbid suspension caused a rapid turbidity decay to initial values, confirming the disassembly of the coacervate micro-droplets (Fig. 2b) due to a further decrease in pH. In control experiments performed in the absence of glucose, the pH remained almost constant (>9.5) and therefore no change in the solution's turbidity was observed (ESI Fig. S6†).

Having established that the catalytic oxidation of glucose by GOx induced a sufficient pH decrease to sequentially trigger the condensation and dissolution of coacervate droplets, we then sought to demonstrate programmable behavior by investigating the outcome of a single-step addition of increasing amounts of glucose fuel to a clear GOx/DEAE-dextran solution prepared at pH ∼ 10. At low glucose concentrations (∼0.6 mM), the turbidity gradually increased until reaching a plateau value (Fig. 2c), indicating that stable coacervate micro-droplets had formed as the optimal coacervation pH was reached (ESI Fig. S6†). Optical microscopy also revealed the gradual nucleation and growth of droplets that persisted for an extended period (Fig. 2d and ESI Movie 1†). We observed that droplets' growth occurred by both fusion and gradual material uptake from the dilute continuous phase with an average area growth rate of 0.059 ± 0.01 μm^2^ min^−1^ (ESI Fig. S7†).

In comparison, higher glucose concentrations (>0.6 mM) resulted in the transient assembly then dissolution of coacervate micro-droplets, as suggested by the bell-shaped temporal evolution of the turbidity (Fig. 2c and ESI Fig. S8†), indicative of the emergence, growth, decay and disassembly of coacervate droplets as the pH decreased from 10.5 down to <6.5 (ESI Fig. S6†). Significantly, we also observed that the full width at half maximum turbidity, *τ*_1/2_, decreased mono-exponentially with the added glucose concentration (Fig. 2f), as expected from the kinetics of GOx-mediated pH decrease (ESI Fig. S9†), indicating that the lifetime of coacervate droplets could be fine-tuned by the amount of added substrate. Optical microscopy further confirmed the transient assembly then dissolution of coacervate micro-droplets at high glucose concentration (Fig. 2e and ESI Movie 2†). Interestingly, at even higher glucose concentrations (5 mM), transient cycles of enzyme-driven spontaneous coacervation could be established by repeated additions of NaOH after droplets had dissolved (to re-increase the pH above 9) until all glucose had been consumed (Fig. 2g). Taken together, these results demonstrate the self-induced biocatalytic condensation and dissolution of enzyme-rich coacervate droplets, together with temporal programmability depending on substrate turnover. Notably, we show that a single-enzyme system suffices to achieve reversible coacervate assembly, provided the enzymatic reaction allows to navigate across the coacervation phase diagram (here, by monotonically altering the net charge and charge density of the polyions).

### Enzyme-driven multiphase droplet organization *via* dynamic polyion self-sorting

We last explored the possibility to use our self-triggered enzyme-rich coacervate platform in more complex environments. *In vivo*, intracellular membraneless organelles evolve in a crowded mixture of components. Studies have shown that these biomolecular condensates can organize into a hierarchical, multi-layered organization, which has been suggested to facilitate the coordination of biochemical reactions in cells.^4,5^ The formation of multiphase complex coacervate droplets has also been recently reported in mixtures of polyelectrolytes at thermodynamic equilibrium *in vitro*.^30–32^ We here sought to expand such hierarchical droplet organization and increase their functional complexity by demonstrating the formation of dynamic multiphase droplets under non-equilibrium enzymatic control.

**Fig. 3:**
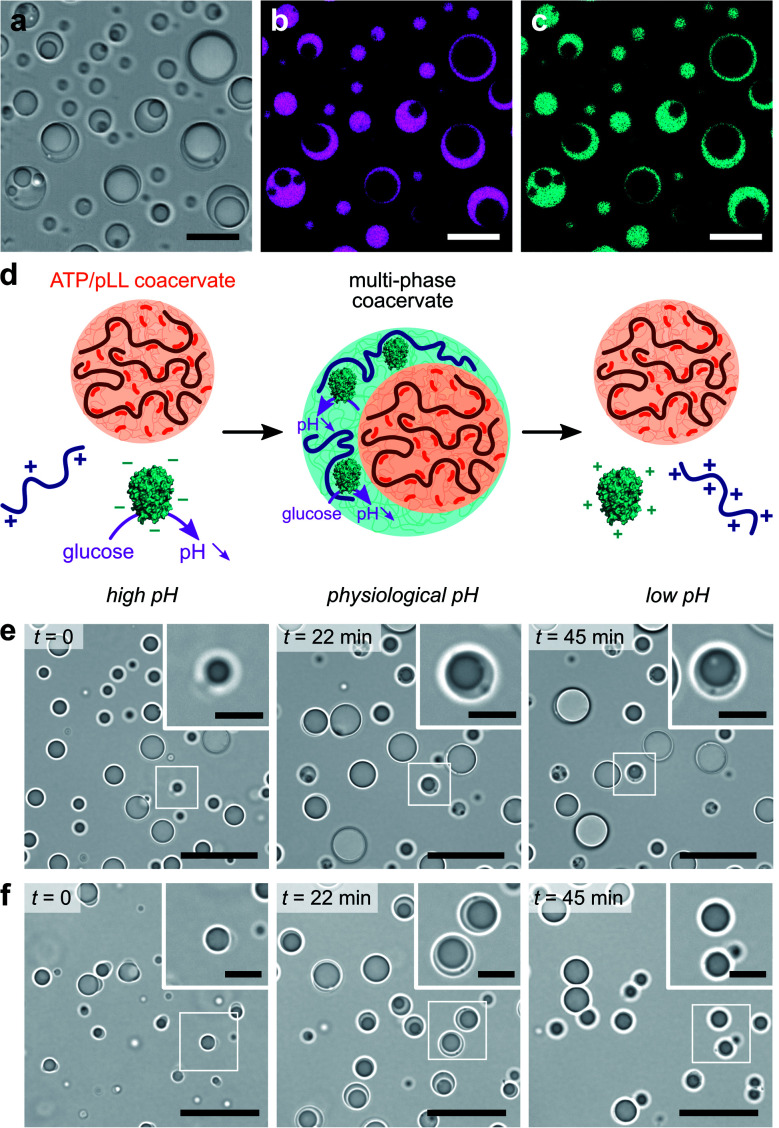
(a–c) Optical (a) and confocal fluorescence (b and c) microscopy images of multiphase ATP/pLL-in-GOx/DEAE-dextran coacervate micro-droplets doped with RITC-GOx (b, red fluorescence) and FITC-DEAE-dextran (c, green fluorescence) in phosphate buffer (2.5 mM, pH 7.4). False coloring to magenta and cyan was used, respectively. Scale bars, 20 μm. (d) Schematic representation of GOx-mediated dynamic formation of multiphase coacervate droplets. At low glucose concentration, stable multiphase droplets are formed as the pH stabilizes to physiological values while higher glucose turnover gives rise to a transient multiphase droplet organization. (e and f) Optical microscopy snapshots of ATP/pLL/GOx/DEAE-dextran mixtures produced at pH 10.2 at different times after addition of 25 mM (e) or 100 mM (f) glucose, showing the formation of stable or transient multiphase coacervate droplets, respectively. Scale bars, 20 μm. Insets show zoomed areas (white box). Scale bars, 5 μm.

We used our GOx/DEAE-dextran droplets in conjunction with coacervates assembled from adenosine triphosphate (ATP) and poly-l-lysine (pLL) as a basis for our dynamic multiphase complex coacervate micro-droplets. Stable multiphase droplets were first successfully formed at equilibrium by mixing equal volumes of suspensions of each of the coacervate droplets prepared separately at their optimal ratio at pH 7.4 (phosphate buffer, 2.5 mM; see Methods). Optical microscopy images confirmed the formation of two-phase droplets showing a smooth interface (Fig. 3a), typical of coexisting liquid phases.^30–32,51^ Both the outer and inner domains behaved as liquids, as demonstrated by their coalescence and ability to engulf other multiphase droplets (ESI Fig. S10†). Confocal fluorescence microscopy further revealed that RITC-GOx and FITC-DEAE-dextran co-localized in the outer phase (Fig. 3b and c), while FITC-labelled pLL selectively localized in the inner phase (ESI Fig. S11†). The multiphase droplets could therefore be described as an outer GOx/DEAE-dextran phase surrounding an inner droplet made from ATP and pLL. This hierarchical organization was likely driven by differences in the surface tension of the two phases, with the inner ATP/pLL coacervates presumably exhibiting a higher interfacial tension than the GOx/DEAE-dextran droplets.^30^ In addition, ATP/pLL coacervates were more resistant to salt than GOx/DEAE-dextran droplets (ESI Fig. S4†), which was consistent with previous studies showing that the inner phase of multiphase coacervates disassembled at higher salt concentrations compared to the outer phase.^30^ Significantly, at high (pH > 8.5) and low (pH < 6.5) pH, or in the absence of DEAE-dextran, single-phase droplets were observed (ESI Fig. S12†), and were attributed to ATP/pLL coacervates since these droplets are stable on a broader pH range (typically, 2 < pH < ∼10.5, ref. 22) compared to GOx/DEAE-dextran droplets.

We finally proceeded to control and program the dynamics of these multiphase coacervate droplets in response to GOx's catalytic activity (Fig. 3d). We herein prepared mixtures of ATP, pLL, GOx and polysaccharide at pH ∼ 10.2, to which we added varying amounts of glucose. We initially observed the presence of single-phase ATP/pLL coacervate droplets (Fig. 3e and f, *t* = 0), as expected at these high pH values, then a second outer liquid phase gradually appeared, associated to the formation of GOx/DEAE-dextran coacervate phase as the solution pH decreased upon GOx catalytic activity (Fig. 3e and f, *t* = 22 min, and ESI Movies 3 and 4†). Depending on the amount of glucose added, we could observe the formation, growth and stabilization (low glucose, Fig. 3e and ESI Movie 3†), or the formation, growth and decay (high glucose, Fig. 3f and ESI Movie 4†) of multiphase coacervate micro-droplets, respectively. Taken together, these observations demonstrate the ability of mixtures of polyions to undergo dynamical self-sorting into multiphase coacervate droplets under non-equilibrium conditions powered by enzyme reactions. Significantly, the amount of fuel supplied to the system controls the formation of stable multiphase complex coacervate droplets or the transient assembly of hierarchical droplets, together with the displacement of GOx molecules between phases.

## Conclusions

In conclusion, we report the self-induced phase separation of an enzyme in the presence of an oppositely charged polyelectrolyte based on enzyme-driven monotonic pH changes. We show that GOx catalytic activity can trigger the formation of stable enzyme-rich coacervate micro-droplets and generate transient coacervates with programmable lifetime depending on the glucose fuel added to the system. We further demonstrate enzyme-driven formation of multiphase complex coacervate droplets *via* spontaneous polyions self-sorting in the presence of a secondary coacervating system. The possibility to timely program such dynamical behavior opens perspectives for the realization of controllable synthetic membraneless organelles as it provides a simple self-mediated biochemical approach to control the compartmentalization of bio-catalytically active molecules.

## Experimental

### Materials

The following chemicals were purchased from Sigma-Aldrich and used as received: glucose oxidase (GOx) from *Aspergillus niger*, diethylaminoethyl-dextran (DEAE-dextran, *M*_w_ = 500 kDa), rhodamine isothiocyanate (RITC), fluorescein isothiocyanate (FITC), fluorescein isothiocyanate labelled diethylaminoethyl-dextran (FITC-DEAE-dextran, *M*_w_ = 70 kDa), potassium carbonate, monopotassium and dipotassium phosphate, toluene, sodium hydroxide, hydrochloric acid (37%), bovine serum albumin (BSA, heat shock fraction, >98%), α-d-glucose (96%), and poly-l-lysine hydrobromide (pLL, (C_6_H_12_N_2_O)_*n*_, 4–15 kDa, monomer *M*_w_ = 208.1 g mol^−1^). Adenosine 5-triphosphate disodium salt hydrate (ATP, C_10_H_14_N_5_Na_2_O_13_P_3_, 551.1 g mol^−1^) was purchased from Carbosynth Limited and 3-[methoxy(polyethyleneoxy)propyl]trimethoxysilane (90%, 6–9 PE units) was purchased from abcr GmbH, Gute Chemie.

### Preparation of stock solutions

Milli-Q water was used to prepare aqueous stock solutions of DEAE-dextran (1.8 mg mL^−1^, corresponding to 8.9 mM average monomer concentration, pH = 7.4), FITC-DEAE-dextran (1.8 mg mL^−1^, pH = 7.4), GOx (6 mg mL^−1^, corresponding to 75 μM monomeric GOx concentration, pH = 7.4), glucose (1 M), pLL (100 mM monomer concentration, corresponding to 14.4 mg mL^−1^, pH = 8.0), ATP (50 mM nucleotide concentration, corresponding to 50.7 mg mL^−1^, pH = 8.0) and phosphate buffer (50 mM, pH = 7.4). The pH of all stocks was adjusted using either NaOH (0.1 M) or HCl (0.1 M). The concentration of GOx in the stock solution was checked by UV-vis spectroscopy (Varian CARY 100 Bio) at 280 nm using an extinction coefficient of 1.67 mL mg^−1^ cm^−1^ (ref. 49) and a molecular weight of 80 000 g mol^−1^. All polymer, protein and mononucleotide stock solutions were stored at −20 °C until use.

### Preparation of fluorescently labelled GOx

A GOx solution (4.0 mg mL^−1^) was prepared by dissolving the freeze-dried protein powder in 1 mL of 0.5 M carbonate buffer at pH = 9.0. An aliquot of a freshly-prepared anhydrous DMSO solution of RITC (10.0 mg mL^−1^) was added drop-wise to the protein solution at a final fluorophore : protein molar ratio of 10 : 1. The reaction mixture was kept at room temperature in the dark for 4 hours, then purified by size exclusion chromatography using a Sephadex G-25 resin (Sigma-Aldrich) eluted with Milli-Q water. The concentration of the fluorescently-labelled proteins in the collected fractions was determined by UV-visible spectrophotometry using the relationship: [protein] = (*A*_280_ − *w* × *A*_max,dye_)/*ε*_protein_, where *A*_280_ and *A*_max,dye_ were the absorbances at 280 nm and at the maximum of absorption of the fluorophore respectively (552 nm for RITC), *w* the correction factor to account for the dye absorption at 280 nm (0.34 for RITC), and *ε*_protein_ the extinction coefficient of the protein (1.67 mL mg^−1^ cm^−1^ for GOx). The dye : protein final molar ratio was determined from the ratio (*A*_max,dye_/*ε*_dye_)/([protein] (mg mL^−1^)/*M*_GOx_), where *ε*_dye_ was the molar extinction coefficient of the dyes at their maximum of absorption (65 000 mol^−1^ L cm^−1^ for RITC), and *M*_GOx_ the molar mass of the protein (80 000 g mol^−1^ for a GOx monomeric unit). Typically, the average dye : protein molar ratio was *ca.* 4 : 1. The RITC-GOx stock solution was split into aliquots and stored at −20 °C until use.

### Preparation of fluorescently labelled poly-l-lysine

A pLL solution (4.0 mg mL^−1^) was prepared by dissolving the freeze-dried polypeptide powder in 1 mL of 0.1 M carbonate buffer at pH = 9.5. An aliquot of a freshly-prepared anhydrous DMSO solution of FITC (10.0 mg mL^−1^) was added drop-wise to the pLL solution at a final fluorophore : pLL chain molar ratio of 1 : 1. The reaction mixture was kept at room temperature in the dark for 4 hours, then purified by multiple washing steps with water using a centrifugal filter (Millipore, Amicon Ultra, MWCO 10 kDa) to remove any unreacted FITC. The FITC-pLL stock solution was split into aliquots and stored at −20 °C until use.

### Passivation of glass coverslips

Glass coverslips were passivated to limit coacervate wetting. Ethanol-rinsed glass coverslips were first incubated for 48 hours in a toluene solution containing 5 wt% of 3-[methoxy(polyethyleneoxy)propyl]trimethoxysilane. Coverslips were subsequently rinsed with toluene, ethanol, and water, and then immersed in an aqueous BSA solution (10 wt%) for another 24 hours, washed with water, dried with compressed air, then assembled into a capillary chamber with a UV-curing glue.

### Phase behaviour of GOx and DEAE-dextran at thermodynamic equilibrium

The influence of the protein and polysaccharide concentration, pH and ionic strength on the formation of coacervates micro-droplets was investigated by monitoring changes in the absorbance at *λ* = 700 nm.

The optimal protein : polyelectrolyte ratio was determined at pH 7.4 on 25 μL of GOx/DEAE-dextran solutions produced at fixed GOx (0.25 mg mL^−1^) and varying DEAE-dextran (0–0.15 mg mL^−1^) concentrations in phosphate buffer (2.5 mM) by mixing in Milli-Q water aliquots of aqueous stock solutions of GOx (6 mg mL^−1^), phosphate buffer (50 mM), and DEAE-dextran (1.8 mg mL^−1^). GOx/DEAE-dextran solutions were prepared in a similar way in the presence of NaCl (10–100 mM final concentrations) to assess the influence of the ionic strength on phase separation. The absorbance of each sample was measured in a 384-well plate (Falcon, flat bottom) using a microplate spectrophotometer (Molecular Devices). All experiments were performed in triplicate and the average values and standard deviations reported.

The pH range of complex coacervation was determined at fixed GOx (0.20, 0.25 and 0.35 mg mL^−1^) and DEAE-dextran (0.03, 0.04, 0.05 mg mL^−1^) concentrations. 200 μL of GOx/DEAE-dextran solutions were prepared in a UV/vis plastic cuvette by mixing aliquots of stock solutions of GOx (6 mg mL^−1^) and DEAE-dextran (1.8 mg mL^−1^) in Milli-Q water, then the pH was adjusted to pH = 10.0 using NaOH (0.1 M), and subsequently dropped by gradual additions of HCl (0.05 M). After each HCl addition, the pH was measured using a calibrated pH meter (Mettler Toledo) equipped with a microelectrode (SI Analytics), and the absorbance of the solution was monitored at 700 nm on a UV-vis spectrometer (Ocean Optics).

DEAE-dextran titration was performed on a 3.6 mg mL^−1^ DEAE-dextran solution prepared in 4 mL Milli-Q water. The pH was initially adjusted to 11.0 using NaOH to ensure that the polysaccharide was fully deprotonated, then HCl (0.1 M) was added stepwise (2 μL steps) as titrant using a micro-pipette and the change in pH was measured using a calibrated pH meter (Mettler Toledo) equipped with a microelectrode (SI Analytics). The exact amount of positive charges was calculated using the volume and molarity of titrant, and the molar mass of each monomer, as detailed in ESI Note 1.† The charge of DEAE-dextran was then compared to that of GOx (see ESI Note 1†).

The amount of GOx sequestered in the polyelectrolyte-rich phase at pH 7.4 was determined by preparing 200 μL of a GOx/DEAE-dextran solution (0.25 mg mL^−1^ GOx, 0.04 mg mL^−1^ DEAE-dextran, 2.5 mM phosphate buffer). The droplets suspension was incubated at room temperature for 15 min, then centrifuged at ∼20 000 × *g* for 15 min to separate the dense coacervate phase from the dilute supernatant. The supernatant solution was removed by pipetting, and the remaining dense coacervate phase was dissolved in 200 μL of a 1 M NaCl solution, then the concentration of GOx in each phase was measured by UV-vis spectroscopy.

### Observation of GOx/DEAE-dextran coacervate micro-droplets

A freshly made GOx/DEAE-dextran solution (0.25 mg mL^−1^ GOx, 0.04 mg mL^−1^ DEAE-dextran) was adjusted to pH 5.5 or 9.5 using HCl (0.1 M) or NaOH (0.1 M) solutions; or prepared at pH 7.4 using phosphate buffer (2.5 mM). Samples were imaged *ca.* 15 minutes after incubation by loading an aliquot of the solution into a passivated capillary chamber (see above). When coacervates formed, the droplets were left to settle for *ca.* 2 minutes on the glass coverslip before imaging. Optical microscopy imaging was performed on a Leica DMI 4000B inverted microscope equipped with a ×63 oil immersion lens (HCX PL APO, 1.4 NA) using the MicroManager software. Images were processed using ImageJ.

To monitor the fusion behaviour of the droplets, a freshly prepared GOx/DEAE-dextran droplets suspension (0.25 mg mL^−1^ GOx, 0.04 mg mL^−1^ DEAE-dextran, 2.5 mM phosphate buffer, pH 7.4) was rapidly loaded into a custom-made passivated capillary chamber. Several coalescence events between contacting droplets could then be observed in real time by optical microscopy imaging. Movies of the coalescence process were acquired on the Leica DMI 4000B inverted microscope equipped with a ×63 oil immersion lens (HCX PL APO, 1.4 NA) using the MicroManager software. The aspect ratio (long axis, *L*, to short axis, *l*) of 10 individual droplets undergoing coalescence was measured as a function of time using ImageJ. The data was fitted to a mono-exponential decay function using OriginLab to determine the relaxation time *τ*: *L*/*l* = *a* + *b* exp(−*t*/*τ*); and the characteristic length scale, *R*, of the droplets was the measured radius after coalescence. The relaxation time, *τ*, is expected to be directly proportional to the characteristic length scale, *R*, of the droplets according to the relation: *τ* ≈ (*η*/*γ*) × *R* (from ref. 50), which gives the inverse capillary viscosity as the ratio of the viscosity of the droplets, *η*, to surface tension, *γ*.

To determine the localization of coacervate components, GOx/DEAE-dextran micro-droplets were doped with RITC-GOx (0.1 μM final concentration) and FITC-DEAE-dextran (0.008 mg mL^−1^ final concentration) by first mixing an aliquot of the fluorescent GOx stock solution (10 μM) with GOx (0.25 mg mL^−1^ final GOx concentration), then adding DEAE-dextran mixed with FITC-DEAE-dextran (4 : 1 DEAE-dextran to FITC-DEAE-dextran molar ratio, 0.04 mg mL^−1^ final DEAE-dextran concentration). The droplets were loaded into a capillary slide and left to settle for 2 minutes before being imaged by confocal fluorescence microscopy on a Leica SP2 confocal laser scanning microscope attached to a Leica DMI RE2 inverted microscope using a ×63 oil immersion lens (HCX PL APO, 1.4 NA). Excitation (and emission) wavelengths were set to 488 nm (emission: 500–550 nm) and 543 nm (emission: 550–650 nm) to monitor FITC-DEAE-dextran and RITC-GOx fluorescence, respectively. Images were processed using ImageJ.

### Enzyme-mediated condensation/dissolution of GOx/DEAE-dextran coacervate micro-droplets

The kinetics of GOx/DEAE-dextran coacervate droplets assembly/disassembly in response to GOx activity was first monitored by turbidity measurements at 25 °C after sequential or single-step addition of glucose. Turbidity measurements were carried out at 700 nm on a microplate spectrophotometer (Molecular Devices) using a 384-well plate (Falcon, flat bottom). A freshly made GOx/DEAE-dextran solution (0.25 mg mL^−1^ GOx, 0.04 mg mL^−1^ DEAE-dextran) was adjusted to pH 10.2 with NaOH (0.1 M) to produce a clear solution of disassembled droplets. For the sequential glucose addition experiment, 3 μL of a 10 mM glucose stock solution were first added to the GOx/DEAE-dextran solution (final glucose concentration of 0.6 mM; total final volume of 50 μL) and absorption values were recorded every minute for 80 min. At *t* = 80 min, another 3 μL of a 10 mM glucose stock solution were added and absorption values recorded every minute for another 80 min. For single-step glucose additions, a fixed amount of glucose (0 mM to 2 mM final concentration at 0.2 mM intervals) was added to the GOx/DEAE-dextran solution (total final volume of 50 μL). The absorbance of each sample was then measured every minute for 160 min. Cycles of transient coacervates assembly were monitored on 50 μL of GOx/DEAE-dextran solution adjusted to pH 10 and supplied with 5 mM glucose (final concentration). Absorption values were recorded every minute for 60 minutes, then 0.5 μL of 1 M NaOH was added (10 mM NaOH final concentration) and values recorded for another ∼45 min. The process was repeated until the absorption did not re-increase spontaneously after NaOH addition (indicating that all glucose had been consumed). All turbidity experiments were performed in triplicate and the average values and standard deviations reported.

We monitored the time-dependent evolution of the solution's pH after glucose addition as follows. A freshly made GOx/DEAE-dextran solution (0.25 mg mL^−1^ GOx, 0.04 mg mL^−1^ DEAE-dextran) was adjusted to pH 10.2 with NaOH (0.1 M) to produce a clear solution of disassembled droplets. To this mixture were added either 1.2 μL of a 100 mM glucose stock solution (total final volume of 200 μL; final glucose concentration of 0.6 mM), or 2.8 μL of a 100 mM glucose stock solution (total final volume of 200 μL; final glucose concentration of 1.4 mM), and the pH was monitored after ∼30 s of equilibration using a calibrated pH meter (Mettler Toledo) equipped with a microelectrode (SI Analytics) under continuous gentle magnetic stirring. pH values were recorded every minute from *t* = 0 to *t* = 90 min. Control experiment without any added glucose was also performed.

Time-dependent optical microscopy of coacervate assembly/disassembly after glucose addition was performed as follows. A freshly prepared GOx/DEAE-dextran droplets suspension (0.40 mg mL^−1^ GOx, 0.064 mg mL^−1^ DEAE-dextran) was adjusted to pH 10.2 and supplied with glucose (0.5 mM or 25 mM final concentration; total final volume of 200 μL), then 5 μL of the suspension were rapidly loaded into a custom-made passivated capillary chamber that was hermetically sealed with UV-curing glue. The sample preparation took *ca.* 2 min after glucose addition. We here used higher GOx (0.40 mg mL^−1^) and DEAE-dextran (0.064 mg mL^−1^) concentrations compared to turbidity measurements (0.25 mg mL^−1^ GOx, 0.04 mg mL^−1^ DEAE-dextran) to form larger droplets that were easier to observe, but we kept the GOx : DEAE-dextran ratio constant. To accelerate the transient coacervate formation process, we also used a higher final glucose concentration (25 mM) compared to turbidity experiments (2 mM). Optical microscopy images of the samples were then acquired every 10 s for 45 min on a Leica DMI 4000B inverted microscope equipped with a ×63 oil immersion lens (HCX PL APO, 1.4 NA) using the MicroManager software. Images were processed using ImageJ.

### Multiphase coacervate preparation and characterization at thermodynamic equilibrium

Each coacervate phase was first prepared separately as follows. 10 μL of a GOx/DEAE-dextran coacervate droplets suspension was produced at 4.4 mg mL^−1^ GOx, 0.70 mg mL^−1^ DEAE-dextran and 2.5 mM phosphate buffer final concentrations by mixing in Milli-Q water aliquots of aqueous stock solutions of GOx (6 mg mL^−1^), phosphate buffer (50 mM), and DEAE-dextran (1.8 mg mL^−1^). Similarly, 10 μL of ATP/pLL coacervate droplets suspension was produced at 10 mM ATP (corresponding to 5.1 mg mL^−1^), 10 mM pLL (monomer concentration, corresponding to 1.4 mg mL^−1^) and 2.5 mM phosphate buffer final concentrations by mixing in Milli-Q water aliquots of aqueous stock solutions of ATP (50 mM), pLL (100 mM) and phosphate buffer (50 mM). The GOx/DEAE-dextran suspension was then added to the ATP/pLL suspension at 1 : 1 volume ratio (total final volume of 20 μL) to give final GOx and DEAE-dextran concentrations of 2.2 mg mL^−1^ and 0.35 mg mL^−1^, respectively, and the obtained turbid suspension was gently mixed. These higher concentrations compared to single-phase droplets were required to observe a sufficiently thick outer coacervate layer in multiphase droplets, presumably due to the lower GOx/DEAE-dextran coacervation efficiency in the presence of ATP and pLL polyions that increased the ionic strength of the solution. Samples were imaged either rapidly (to observe fusion events of the inner droplets) or after *ca.* 5 minutes by loading an aliquot of the solution into a custom-made passivated capillary chamber (see above). Optical microscopy imaging was performed on a Leica DMI 4000B inverted microscope equipped with a ×63 oil immersion lens (HCX PL APO, 1.4 NA) using the MicroManager software. Images were processed using ImageJ.

To determine the localization of the coacervate components, GOx/DEAE-dextran micro-droplets were doped with RITC-GOx (0.25 μM final concentration) and FITC-DEAE-dextran (0.07 mg mL^−1^ final concentration) by first mixing an aliquot of the fluorescent GOx stock solution (10 μM) with GOx (2.2 mg mL^−1^ final GOx concentration), then adding DEAE-dextran mixed with FITC-DEAE-dextran (4 : 1 DEAE-dextran to FITC-DEAE-dextran molar ratio, 0.35 mg mL^−1^ final DEAE-dextran concentration). Control images were also acquired in the absence of DEAE-dextran. Alternatively, multiphase droplets were prepared using ATP/pLL droplets doped with FITC-pLL (2 mM, *i.e.* 0.25 mg mL^−1^ final concentration). Multiphase droplets were then formed as described above then loaded into a capillary slide and left to settle for 2 minutes before being imaged by confocal fluorescence microscopy on a Leica SP2 confocal laser scanning microscope attached to a Leica DMI RE2 inverted microscope using a ×63 oil immersion lens (HCX PL APO, 1.4 NA). Excitation (and emission) wavelengths were set to 488 nm (emission: 500–540 nm) and 543 nm (emission: 550–650 nm) to monitor FITC-DEAE-dextran and RITC-GOx fluorescence, respectively. Images were processed using ImageJ.

We assessed the influence of the ionic strength on ATP/pLL coacervate microdroplets as for the GOx/DEAE-dextran coacervates. Briefly, 50 μL of ATP/pLL coacervates were produced at pH 7.4 (10 mM ATP, 10 mM pLL, 2.5 mM phosphate buffer) in the presence of increasing amounts of NaCl (0–400 mM), and the turbidity monitored at 700 nm in a 384-well plate (Falcon, flat bottom) using a microplate spectrophotometer (Molecular Devices). Experiments were performed in triplicate and the average values and standard deviations reported.

### Enzyme-regulated dynamic assembly/disassembly of multiphase droplets

GOx/DEAE-dextran (2.2 mg mL^−1^ GOx, 0.35 mg mL^−1^ DEAE-dextran) and ATP/pLL (10 mM ATP, 10 mM pLL) solutions were freshly prepared separately in Milli-Q water and mixed at 1 : 1 volume ratio. The pH was adjusted to ∼10.2 using NaOH and the solutions supplied with glucose (25 mM or 100 mM final concentration; total final volume of 20 μL), then 5 μL of the solution were rapidly loaded into a custom-made passivated capillary chamber that was hermetically sealed with UV-curing glue. The sample preparation took *ca.* 2 min after glucose addition. Optical microscopy images of the samples were then acquired every 10 s for 57 min on a Leica DMI 4000B inverted microscope equipped with a ×63 oil immersion lens (HCX PL APO, 1.4 NA) using the MicroManager software. Images were processed using ImageJ.

## Author contributions

N. M. designed the project, N. M. and H. K. designed the experiments, H. K. and M. J. S. performed the experiments, N. M. and H. K. analysed the results and wrote the manuscript.

## Conflicts of interest

The authors declare no conflict of interest.

## Supplementary Material

SC-012-D0SC06418A-s001

SC-012-D0SC06418A-s002

SC-012-D0SC06418A-s003

SC-012-D0SC06418A-s004

SC-012-D0SC06418A-s005
