# Supplementary material for: Self-programmed enzyme phase separation and multiphase coacervate droplet organization
Source: Chem Sci. 2021 Jan 25;12(8):2794–802. doi: 10.1039/d0sc06418a (PMC8179374; doi:10.1039/d0sc06418a)
Supplement: SC-012-D0SC06418A-s001 [file SC-012-D0SC06418A-s001.pdf]

Electronic Supplementary Material (ESI) for Chemical Science.
This journal is © The Royal Society of Chemistry 2021

Supporting Information

Self-Programmed Enzyme Phase Separation and Multiphase Coacervate Droplet Organization

Hedi Karoui,[a] Marianne J. Seck,[a] Nicolas Martin[a]*

[a] Univ. Bordeaux, CNRS, Centre de Recherche Paul Pascal, UMR5031, 115 Avenue du Dr. Schweitzer, 33600 Pessac, France

*E-mail: nicolas.martin@crpp.cnrs.fr

1. Supplementary Note 1 : charge rationalization.

Turbidity measurements at varying DEAE-dextran and GOx concentrations (pH 7.4), or at varying pH (fixed DEAE-dextran and GOx concentrations), showed a maximum for a specific ratio (Fig. 1b) or pH (Fig. 1c), respectively, corresponding to optimal coacervation conditions. We sought to determine the ratio of positive vs. negative charges for these conditions by combining charge titration and calculation studies.

Charge titration of DEAE-dextran.
We started by titrating DEAE-dextran to estimate the amount of positive charges on the polymer at each pH (Supplementary Fig. S1a,b, Supplementary Table S1 and Methods). This titration study allowed us to determine:

- the apparent pKa for the strong (pKa_1 = 8.8) and weak (pKa_2 = 5.4) tertiary amines on DEAE-dextran. These values were directly obtained from the titration curve at the half-equivalence point of each amine (as indicated on Supplementary Fig. S1b).
- the fraction of each monomer: i = 0.16 ± 0 (strong amine monomer); j = 0.69 ± 0.01 (neutral glucose monomer); k = 0.15 ± 0.01 (weak amine/quaternary ammonium monomer). These values were obtained from the molar amount of each amine, which was determined from their respective equivalence points (Supplementary Fig. S1b and Supplementary Table S1), together with the molar amount of the neutral glucose monomer. The latter was determined from the known total mass of titrated DEAE-dextran, m_{total}, and the molar amount of each amine using the relationship:

$$n_{glucose} = n_{total} - n_{strong} - n_{weak} = \frac{(m_{total} - n_{strong} \cdot M_{strong} - n_{weak} \cdot M_{weak})}{M_{glucose}} \quad \text{(Eq. S1)}$$

  where "*glucose*", "*strong*" and "*weak*" denote the neutral glucose monomer, the strong amine monomer, and the weak amine/ammonium monomer, respectively; n refers to the molar amount; and M is the molecular weight (M_{strong} = 245 g mol^{-1}, $M_{glucose}$ = 160 g mol^{-1}; M_{weak} = 345 g mol^{-1} according to the chemical structure of DEAE-dextran).
- the average monomer molecular weight of DEAE-dextran: $M_{monomer}$ = 202.4 ± 2.4 g mol^{-1}, based on the fraction of each monomer determined above and their respective molecular weights;
- last, the net charge of a single DEAE-dextran as a function of pH (Supplementary Table S2). For this, we first determined the fraction of ionized nitrogen atoms, f_+, as a function of pH from the titration curve by using the relationship:

$$f_+ = 100. \frac{n_{ammonium} + n_{HCl}}{n_{strong} + n_{weak} + n_{ammonium}} \quad \text{(Eq. S2)}$$

  where $n_{ammonium}$ refers to the total molar amount of weak amine/ammonium monomers, n_{strong} and n_{weak} are the molar amount of strong and weak amine monomer, respectively, and n_{HCl} is the total molar amount of HCl added after each HCl addition (which depends on the pH). By using the average number of monomers on a chain of DEAE-dextran ($N_{DEAE\text{-}dextran\ monomers}$ ~ 2,470, given that $M_{DEAE\text{-}dextran}$ ~ 500,000 g mol^{-1} and $M_{monomer}$ = 202.4 g mol^{-1}), and the fraction of strong amine (i) and weak amine/ammonium monomer (k), we then calculated the pH-dependent net charge of a single DEAE-dextran chain:

$$net\ charge = f_+ \ . \ N_{DEAE-dextran\ monomers} \cdot (i + 2k) \quad \text{(Eq. S3)}$$

Charge calculations for GOx.
The net charge of GOx was computed online using the pdb entry *1cf3* (corresponding to glucose oxidase from *Aspergillus Niger*) with the propKa tool[1,2] at different pH (http://server.poissonboltzmann.org/pdb2pqr). Results of the propKa analysis gave the list of ionisable residues on the protein, together with their apparent pKa (determined by taking into account their local environment), and their fraction of solvent accessible surface area, f_{SASA}. This list is given in Supplementary Table S3.
To calculate the net charge of GOx, we arbitrarily chose not to consider residues with a solvent accessible surface area below 20% (Supplementary Table S3). Since GOx forms a dimer in solution, we

also removed residues located in the contact area between the two GOx subunits in the dimer (Supplementary Table S3). For this purpose, we first aligned two GOx monomers (pdb entry: *1cf3*) onto the two subunits of a GOx dimer (pdb entry: *1gpe*) using the “align” tool on PyMol, then visually investigated on PyMol which of the ionisable residues where located in the contact area between the two GOx subunits.

We then calculated the pH-dependent charge of all remaining ionisable residues with the Henderson-Hasselbalch relationship using their respective pKa. Summing the charges of all residues gave the net charge of GOx as a function of pH (Supplementary Table S2). This analysis also gave an isoelectric point for GOx of 5.02.

Determination of the pH for charge neutralization and comparison to the experimental pH of optimal coacervation.

We finally calculated the product of the net charge on a single polymer or protein molecule, z_i (where *i* stands for DEAE-dextran or GOx) by the concentration of DEAE-dextran chains, $c_{DEAE\text{-}dextran}$, or GOx molecules, c_{GOx}, respectively, for a given concentration of each component, as shown on Supplementary Fig. S1c and reported in Supplementary Table S2 for c_{GOx} = 0.25 mg mL^{-1} and $c_{DEAE\text{-}dextran}$ = 0.04 mg mL^{-1}.

By summing these two products, $\sum_i z_i . c_i = z_{GOx} . c_{GOx} + z_{DEAE-dextran} . c_{DEAE-dextran}$, we were able to determine the pH required for charge neutralization, $\sum_i z_i . c_i = 0$ (Supplementary Fig. S1e,h). This pH was then compared to the optimal coacervation pH obtained by turbidity measurements at varying DEAE-dextran/GOx ratio (Supplementary Fig. S1f,i). These two pH values (experimental and theoretical) agree very well (Supplementary Fig. S1g,j).

2. Supplementary Figures

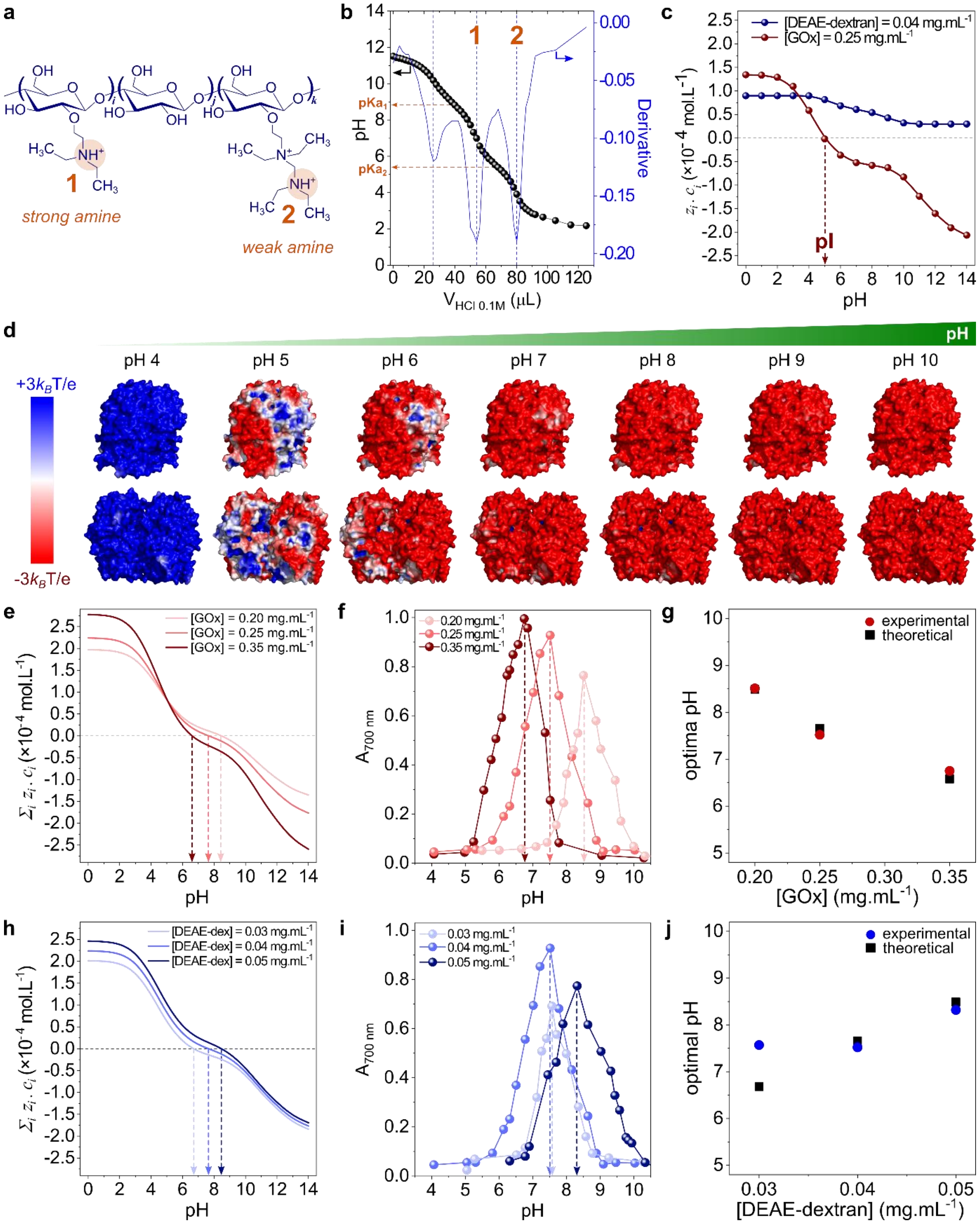

Supplementary Fig. S1. Charge titration and calculation studies. **a**, Chemical structure of DEAE-dextran, showing three types of monomers with respective fractions *i* (strong amine monomer), *j* (neutral glucose monomer) and *k* (weak amine/ammonium monomer). **b**, pH titration of DEAE-dextran (3.6 mg mL^{-1}) in the presence of an excess of sodium hydroxide with a 0.1 M HCl solution (black dots), and associated derivative (blue line), which was used to identify the three equivalence points. The first equivalence point corresponds to the neutralization of the excess sodium hydroxide added, while the

following two equivalence points correspond to the titration of the strong and weak amines, respectively. The apparent pKa of both amines is determined at half of their respective equivalence points. **c**, Product of the net charge of a single polymer or protein molecule, z_i, by its concentration, c_i, (see Supplementary Note 1 for details) for 0.04 mg mL^{-1} DEAE-dextran (blue) and 0.25 mg mL^{-1} GOx (red). The protein isoelectric point (pI) can be determined at $z_{GOx}.c_{GOx}$ = 0. **d**, Images showing the electrostatic surface potential of a single GOx dimer at varying pH (scale bar: ± $3k_BT/e$). The potential was calculated using the online Adaptive Poisson-Boltzmann Solver software[3,4] on a GOx monomeric unit (pdb entry: *1cf3*), and visualized on a GOx dimer using PyMol (see Supplementary Note 1 for details). Two different views (side view, top; front view, bottom) of the same dimer are shown. **e**,**h**, Sum of the product of the net charge of GOx, z_{GOx}, by its concentration, c_{GOx}, and the net charge of DEAE-dextran, $z_{DEAE\text{-}dextran}$, by its concentration, $c_{DEAE\text{-}dextran}$ (see Supplementary Note 1 for details), at 0.04 mg mL^{-1} DEAE-dextran and varying GOx concentrations (**e**), and at 0.25 mg mL^{-1} GOx and varying DEAE-dextran concentrations (**h**). The pH of charge neutralization between GOx and DEAE-dextran can be determined from these plots ($\sum_i z_i . c_i = 0$, dotted arrows). **f**,**i**, Plot of the absorbance at 700 nm of mixtures of GOx and DEAE-dextran as a function of the pH at 0.04 mg mL^{-1} DEAE-dextran and varying GOx concentrations (**f**), and at 0.25 mg mL^{-1} GOx and varying DEAE-dextran concentrations (**i**). The optimal pH of complex coacervation can be determined from these plots (maximum turbidity, dotted arrows) **g**,**j**, Comparison of the theoretical pH of GOx/DEAE-dextran charge neutralization and the optimal coacervation pH at fixed DEAE-dextran concentration (0.04 mg mL^{-1}) and varying GOx concentration (**g**), or fixed GOx concentration (0.25 mg mL^{-1}) and varying DEAE-dextran concentration (**j**).

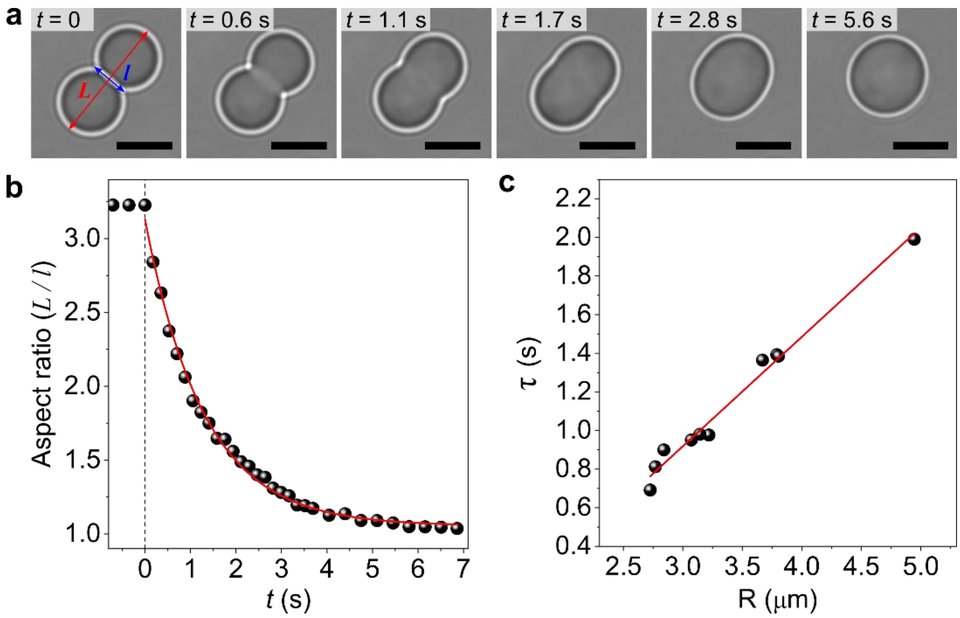

Supplementary Fig. S2. Fusion of GOx/DEAE-dextran coacervate droplets. **a**, Optical microscopy images showing coalescence of two GOx/DEAE-dextran droplets and relaxation to a single spherical micro-droplet over a few seconds. Scale bars, 5 µm. **b**, Plot of the aspect ratio (ratio of major axis, *L*, over minor axis, *l*, as shown in **a**, *t* = 0) of the droplets undergoing coalescence shown in **a** as a function of time. The red curve is a fit to a mono-exponential decay. **c**, Plot of the characteristic time of exponential decay, τ, as a function of the final radius of the droplet after fusion, R. The red linear fit has a slope of 0.56 ± 0.03 s μm^{-1} corresponding to the inverse capillary velocity, η/γ.

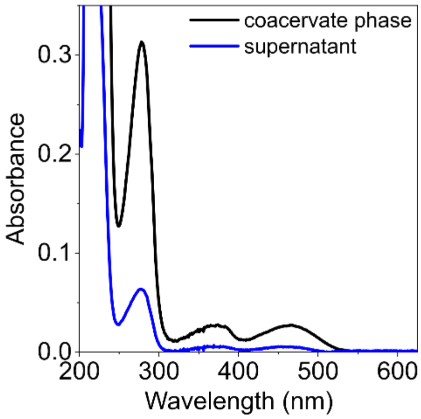

Supplementary Fig. S3. Partitioning of GOx. Absorbance associated to GOx in the bulk coacervate phase (black line) and supernatant (blue line). From these plots, we estimated that the mass fraction of GOx in the bulk coacervate phase represents ~ 80% of the total mass of GOx added.

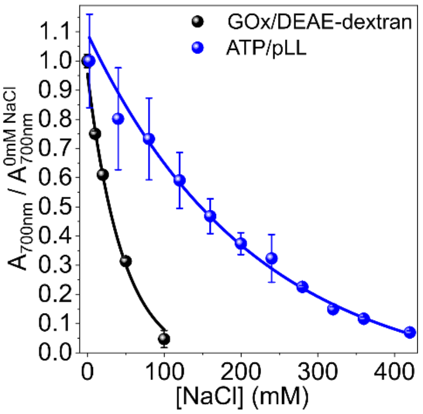

Supplementary Fig. S4. Effect of salt on coacervation. Plot of the absorbance at 700 nm of a mixture of GOx (0.25 mg mL^{-1}) and DEAE-dextran (0.04 mg mL^{-1}), or a mixture of ATP (10 mM) and pLL (10 mM), prepared in phosphate buffer (2.5 mM, pH 7.4) at increasing added sodium chloride, showing the gradual dissolution of coacervate droplets as the ionic strength increases. The lines are a guide to the eye.

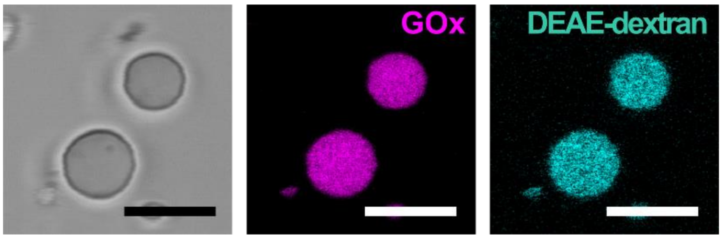

Supplementary Fig. S5. Localization of GOx and DEAE-dextran. Bright-field (left) and confocal fluorescence microscopy images of GOx/DEAE-dextran coacervate micro-droplets doped with RITC-GOx (centre, red fluorescence) and FITC-DEAE-dextran (right, green fluorescent). False colouring to magenta and cyan was used, respectively. Scale bar, 20 µm.

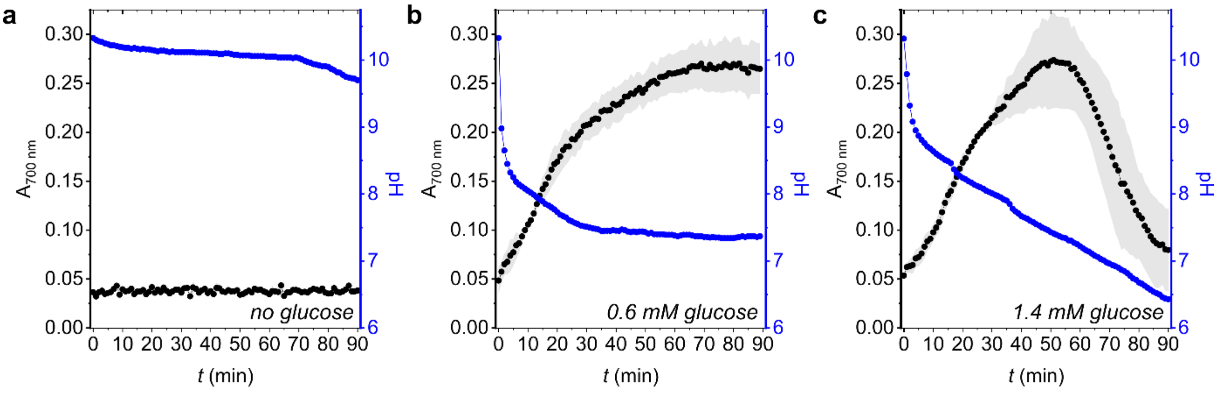

Supplementary Fig. S6. Kinetics turbidity and pH measurements. **a**-**c**, Time-dependent evolution of the turbidity (black dots) and pH values (blue dots) in mixtures of GOx (0.25 mg mL^{-1}) and DEAE-dextran (0.04 mg mL^{-1}) prepared at pH ~ 10.2 in the absence of glucose (**a**), and after addition of 0.6 mM (**b**) or 1.4 mM (**c**) glucose (final concentration).

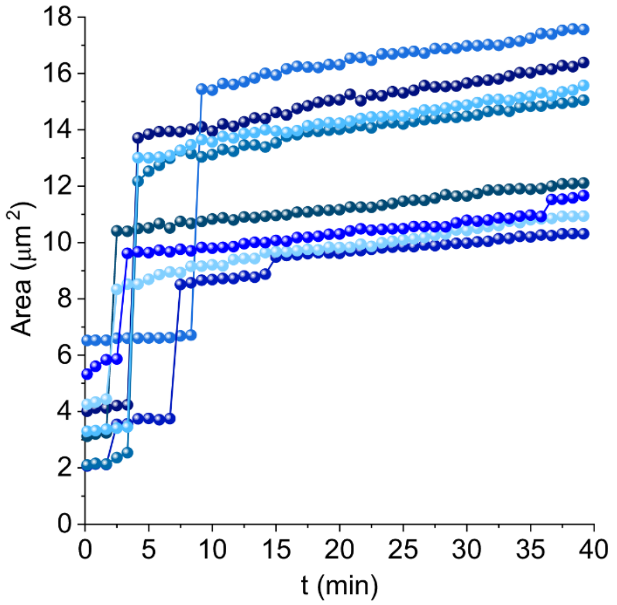

Supplementary Fig. S7. Time dependent droplet growth. Plot of the time-dependent growth of 8 individual coacervate microdroplets (reported as the projected surface area) from a mixture of GOx (0.40 mg mL^{-1}) and DEAE-dextran (0.064 mg mL^{-1}) prepared at pH ~ 10.2 and supplied with 0.6 mM glucose. Droplet growth occurred by both fusion (corresponding to the jumps in area) and gradual material uptake (progressive area growth) from the dilute continuous phase with an average growth rate of 0.059 ± 0.01 μm^2 min^{-1}.

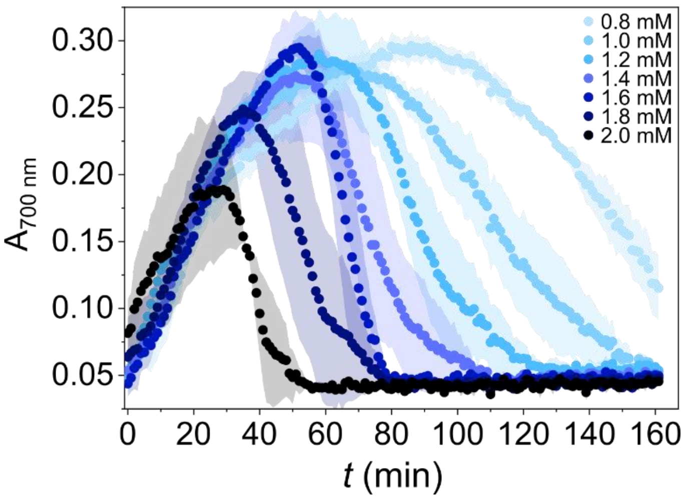

Supplementary Fig. S8. Time-dependent evolution of the absorbance at 700 nm of a solution of GOx (0.25 mg.mL^{-1}) and DEAE-dextran (0.04 mg.mL^{-1}) produced at pH 10.2 after the single-step addition of varying final glucose concentrations, as indicated. The colored area represents error as the standard deviation of three independent repeats.

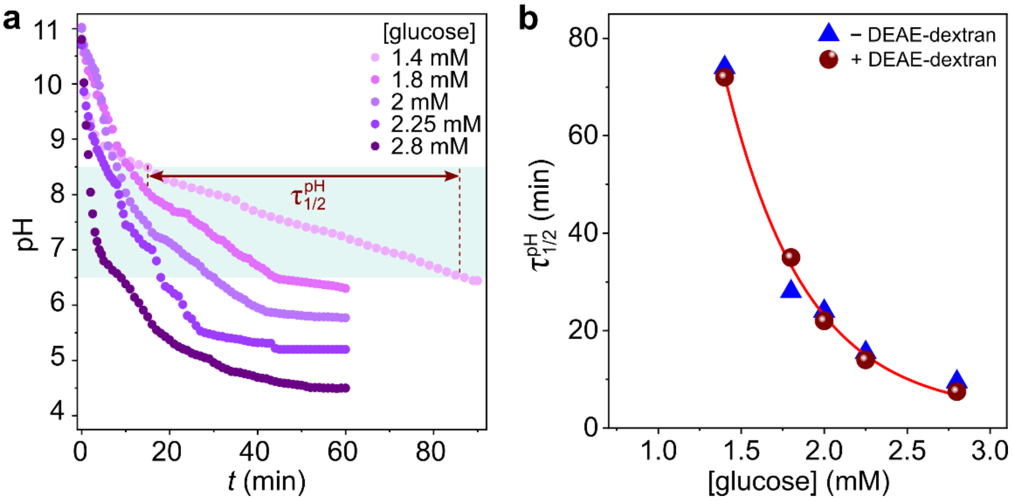

Supplementary Fig. S9. Kinetics of GOx-induced pH decrease**. a,** Time-dependent evolution of the pH of a solution of GOx (0.25 mg.mL^{-1}) and DEAE-dextran (0.04 mg.mL^{-1}) produced at pH > 10 after the single-step addition of varying final glucose concentrations, as indicated. The colored area represents the pH domain of coacervate existence (8.5 < pH < 6.5). $\tau^{\mathrm{pH}}_{1/2}$ denotes the time required for the pH to drop from 8.5 to 6.5. **b**, Evolution of $\tau^{\mathrm{pH}}_{1/2}$ as defined in **a** as a function of the glucose concentration, in the absence (blue triangle) or presence (dark red circles) of DEAE-dextran. The red line represents a mono-exponential fit of the data with DEAE-dextran. The characteristic glucose concentration of this fit is $[\mathrm{glucose}]_{\mathrm{pH}}$ = 0.49 mM, which is comparable to the value obtained from the mono-exponential fit of the droplets' lifetime shown in Figure 2f ($[\mathrm{glucose}]_{\mathrm{lifetime}}$ = 0.51 mM). This correlation confirms that the mono-exponential decay of coacervates' lifetime as a function of the glucose concentration correlates to the kinetics of GOx-mediated pH decrease. The kinetics of GOx-mediated pH decrease is similar in the absence of DEAE-dextran (blue triangles).

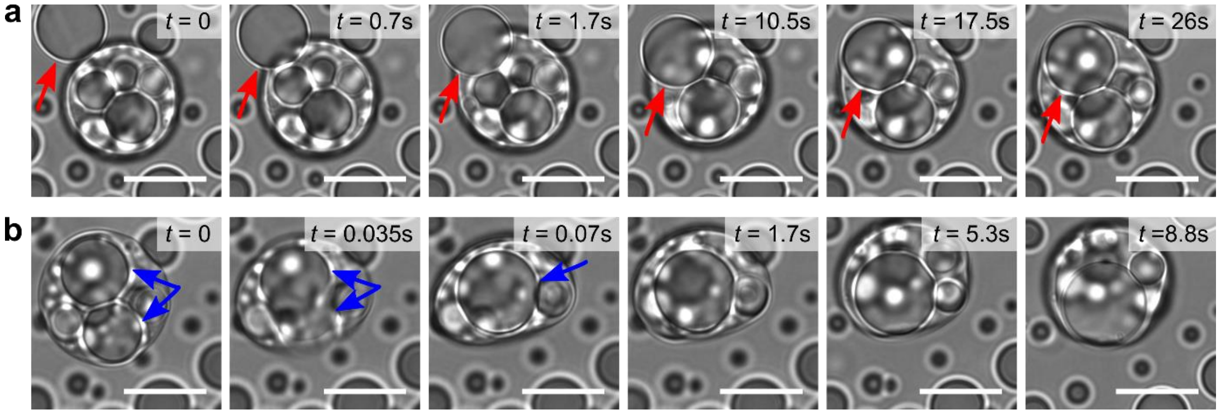

Supplementary Fig. S10. Liquid-like behaviour of multiphase droplets. **a**,**b**, Optical microscopy snapshots of engulfment of an ATP/pLL coacervate droplet (red arrow) by a multiphase droplet (**a**), and fusion and relaxation of two ATP/pLL coacervates (blue arrows) embedded within a GOx/DEAE-dextran droplet (**b**).

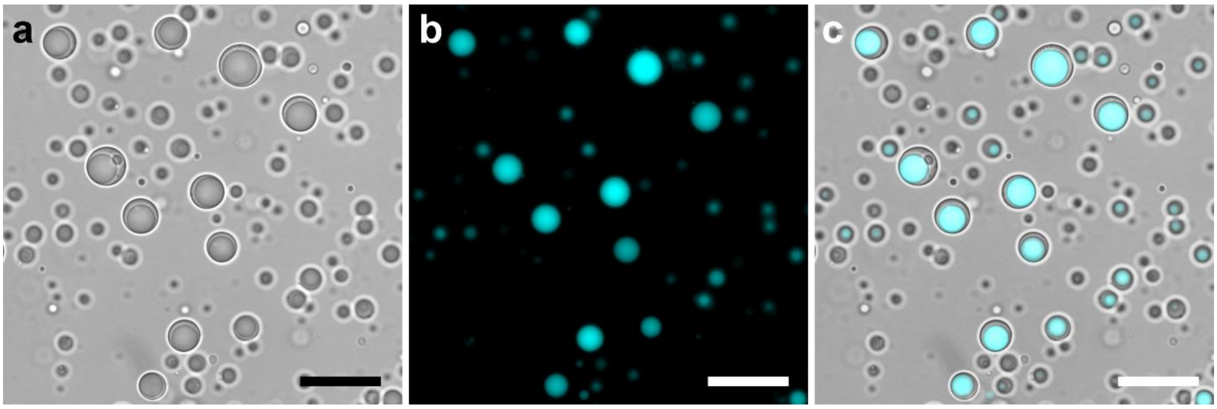

Supplementary Fig. S11. Localization of fluorescently-labelled pLL in multiphase droplets**.** Optical (**a**) and confocal fluorescence (**b**) microscopy images of multiphase ATP/pLL-in-GOx/DEAE-dextran coacervate micro-droplets doped with FITC-pLL (**b**, green fluorescence) in phosphate buffer (2.5 mM, pH 7.4). False coloring to cyan was used. **c** shows an overlay of bright-field and fluorescence imaging, confirming that FITC-pLL selectively localizes in the inner phase of multiphase droplets. Scale bars, 20 µm

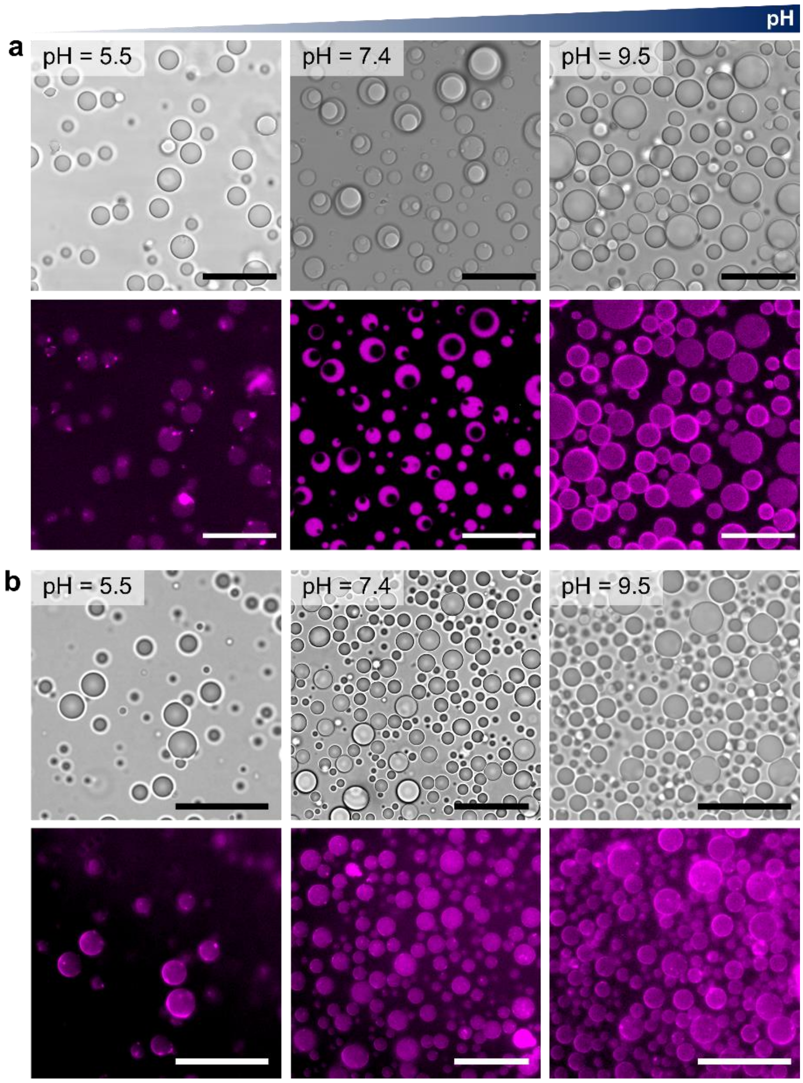

Supplementary Fig. S12. a, Optical (top) and confocal fluorescence (bottom) microscopy images of ATP/pLL/GOx/DEAE-dextran solutions doped with RITC-GOx (bottom, red fluorescence) at pH 5.5, in phosphate buffer (2.5 mM, pH 7.4), and at pH 9.5, respectively, showing the presence of ATP/pLL single phase droplets at pH 5.5 and pH 9.5 and multiphase ATP/pLL-in-GOx/DEAE-dextran droplets at pH 7.4. Accumulation of RITC-GOx at the droplets interface is observed at pH 9.5. False coloring to magenta was used. Scale bars, 20 µm. **b,** Optical (top) and confocal fluorescence (bottom) microscopy images of ATP/pLL solutions prepared in the presence of GOx doped with RITC-GOx (bottom, red fluorescence) but in the absence of DEAE-dextran at pH 5.5, in phosphate buffer (2.5 mM, pH 7.4), and at pH 9.5, respectively, showing the sequestration of RITC-GOx in single-phase ATP/pLL droplets regardless of the pH but no multiphase organization. False coloring to magenta was used. Scale bars, 20 µm.

3. Supplementary Tables.

Supplementary Table S1. Results from the titration of DEAE-dextran (at a concentration of 3.6 mg mL^{-1}, corresponding to ~ 17.9 mM total monomer concentration, and an average polymer chain concentration of ~ 7.2 µM). The titration was done in triplicate and average values and standard deviations reported.

	Strong amine monomer	Weak amine/ammonium monomer	Glucose monomer	Total DEAE-dextran monomers
n (mmol)	2.8 ± 0.08	2.7 ± 0.2	12.4 ± 0.4	17.9
Fraction (%)	16 ± 0.4	15 ± 1	69 ± 2	100

Supplementary Table S2. Fraction of ionized nitrogen atoms, f_+, on DEAE dextran, and average net charge of a single DEAE-dextran polymer chain and a single GOx monomer as a function of the pH. An example of product of net charge by concentration, $z_i \cdot c_i$, is also given for a GOx concentration of 0.25 mg mL^{-1} and a DEAE-dextran concentration of 0.04 mg mL^{-1}. In this case, the pH for charge neutralization is comprised between 7 and 8 (bold values). Blue and red shading correspond to positive and negative net charge, respectively.

pH	DEAE-dextran chain		GOx monomer	$z_{DEAE\text{-}dextran} \cdot c_{\text{DEAE-dextran}}$	$z_{GOx} \cdot c_{\text{GOx}}$
	f_+ (%)	Net charge	Net charge	(µmol L^{-1})	(µmol L^{-1})
0	100	+1120	+43	+90	+134
1	100	+1120	+43	+90	+133
2	100	+1120	+41	+90	+130
3	100	+1120	+35	+90	+110
4	100	+1120	+19	+90	+59
5	91.0	+1020	-0.6	+82	-2
6	76.4	+850	-12	+69	-37
7	67.6	+760	-17	**+61**	**-52**
8	60.2	+670	-19	**+54**	**-58**
9	47.5	+530	-20	+43	-63
10	35.5	+400	-27	+32	-83
11	33.1	+370	-40	+30	-124
12	33.1	+370	-51	+30	-160
13	33.1	+370	-61	+30	-191
14	33.1	+370	-66	+30	-206

Supplementary Table S3. List of the ionizable residues of GOx (pdb entry: *1cf3*) with their apparent pKa and fraction of solvent accessible surface area, f_{SASA}. Grey shading: residues that are buried more than 80% (f_{SASA} < 20%). Blue shading: residues located in the contact area between two GOx subunits in a GOx dimer. These residues were not taken into account for the pH-dependent charge calculation of GOx.

Residue		pKa	f_{SASA} (%)
ASP	11	3.27	100
ASP	14	4.23	100
ASP	21	3.95	65
ASP	57	3.72	95
ASP	64	3.06	75
ASP	70	4.07	100
ASP	77	3.09	48
ASP	120	4.24	73
ASP	134	4.01	100
ASP	177	1.59	80
ASP	180	3.24	100
ASP	181	3.94	100
ASP	195	4.03	100
ASP	203	3.69	28
ASP	208	3.87	52
ASP	222	3.3	93
ASP	227	5.54	0
ASP	315	4	100
ASP	319	3.73	80
ASP	328	5.82	0
ASP	360	3.89	100
ASP	401	3.31	84
ASP	416	5.43	8
ASP	424	3.2	2
ASP	427	9.29	0
ASP	440	3.1	100
ASP	442	3.83	89
ASP	451	5.16	34
ASP	460	6.56	0
ASP	492	3.91	100
ASP	497	3.51	100
ASP	499	2.61	100
ASP	533	3.24	39
ASP	548	4.96	0
ASP	573	3.84	63
ASP	578	3.14	71
GLU	5	4.95	100
GLU	40	4.67	62
GLU	50	8.62	0
GLU	55	5.09	52
GLU	63	5.04	86
GLU	81	4.15	100
GLU	84	4.71	100
GLU	123	3.34	100
GLU	129	4.67	100
GLU	144	2.85	10
GLU	194	4.51	100
GLU	221	4.5	100
GLU	231	4.56	9
GLU	268	4.72	58
GLU	284	4.73	17
GLU	299	8.58	2
GLU	310	4.51	100
GLU	356	4.38	32

Residue		pKa	f_{SASA} (%)
GLU	363	4.52	100
GLU	367	4.62	100
GLU	374	5.89	100
GLU	378	4.23	100
GLU	379	4.13	95
GLU	397	4.95	89
GLU	412	9.11	0
GLU	458	4.35	76
GLU	487	4.38	55
GLU	505	4.73	100
GLU	527	4.04	100
GLU	577	4.81	100
C-	583	3.25	100
HIS	78	3.2	3
HIS	115	4.83	37
HIS	158	3.96	9
HIS	165	7.11	19
HIS	172	6.67	64
HIS	210	6.6	39
HIS	220	6.07	50
HIS	272	6.21	100
HIS	277	5.85	92
HIS	283	5.63	70
HIS	366	5.78	70
HIS	387	5.01	42
HIS	406	6.21	79
HIS	437	4.06	15
HIS	446	4.93	29
HIS	447	6.28	100
HIS	510	6.28	74
HIS	516	3.43	0
HIS	559	2.21	0
CYS	164	99.99	100
CYS	206	99.99	100
CYS	521	13.49	0
TYR	22	13.25	39
TYR	54	11.82	56
TYR	68	12.45	1
TYR	80	14.86	0
TYR	139	12.5	67
TYR	159	10.44	100
TYR	182	12.66	49
TYR	237	10.22	100
TYR	249	11.6	33
TYR	280	10	100
TYR	300	12.83	26
TYR	361	10.35	99
TYR	396	12.67	0
TYR	399	11.55	0
TYR	410	17.03	0
TYR	435	11.36	54
TYR	444	14.59	30
TYR	450	13.91	0
TYR	454	18.37	0

Residue		pKa	f_{SASA} (%)
TYR	483	10.35	96
TYR	496	10.63	71
TYR	506	11.83	56
TYR	509	10.23	100
TYR	515	11.12	22
TYR	539	12.35	51
TYR	565	14.33	0
TYR	579	10.78	98
LYS	13	10.45	100
LYS	116	10.17	82
LYS	152	11.3	100
LYS	187	10.4	100
LYS	201	9.8	78
LYS	202	10.38	86
LYS	252	10.2	59
LYS	273	10.49	100
LYS	282	10.57	100
LYS	306	10.71	100
LYS	364	10.54	100
LYS	372	10.54	100
LYS	441	10.53	100
LYS	526	10.35	100
LYS	570	9.4	50
ARG	18	12.36	100
ARG	37	12.76	50
ARG	58	13.56	98
ARG	95	13.19	78
ARG	113	12.38	0
ARG	145	12.31	100
ARG	147	11.11	47
ARG	176	10.61	6
ARG	196	11.65	62
ARG	225	9.77	0
ARG	230	12.96	52
ARG	239	11.65	71
ARG	263	12.01	71
ARG	335	12.19	70
ARG	337	12.32	100
ARG	383	11.67	51
ARG	400	12.19	93
ARG	433	12.09	0
ARG	472	13.03	60
ARG	512	14.7	2
ARG	537	13.16	65
ARG	545	14.18	19
N+	3	7.77	100

4. Supplementary Movies.

Supplementary Movie 1. Optical microscopy video of a solution of GOx (0.40 $mg.mL^{-1}$) and DEAE-dextran (0.064 $mg.mL^{-1}$) prepared at pH 10.2 after addition of 0.5 mM glucose, showing the gradual nucleation, growth and stabilisation of coacervate micro-droplets. Partial wetting of the droplets is observed towards the end of the movie (loss of spherical shape). Movie is shown at ×100 real-time speed at 10 frames per seconds. Total time in real time was ~ 45 minutes. Scale bar, 10 µm.

Supplementary Movie 2. Optical microscopy video of a solution of GOx (0.40 $mg.mL^{-1}$) and DEAE-dextran (0.064 $mg.mL^{-1}$) prepared at pH 10.2 after addition of 25 mM glucose, showing the gradual nucleation, growth, then decay and dissolution of coacervate micro-droplets. Movie is shown at ×100 real-time speed at 10 frames per seconds. Total time in real time was ~ 45 minutes. Scale bar, 10 µm.

Supplementary Movie 3. Optical microscopy movie of a solution of GOx (2.2 $mg.mL^{-1}$), DEAE-dextran (0.35 $mg.mL^{-1}$), ATP (10 mM) and pLL (10 mM) prepared at pH 10.2 after addition of 25 mM glucose. Initially, single-phase ATP/pLL coacervate droplets are present, then an outer GOx/DEAE-dextran liquid phase gradually forms and grows around them to produce stable multiphase coacervate droplets. Movie is shown at ×100 real-time speed at 10 frames per seconds. Total time in real-time speed was ~ 57 minutes. Scale bar, 20 µm.

Supplementary Movie 4. Optical microscopy movie of a solution of (2.2 $mg.mL^{-1}$), DEAE-dextran (0.35 $mg.mL^{-1}$), ATP (10 mM) and pLL (10 mM) prepared at pH 10.2 after addition of 100 mM glucose. Initially, single-phase ATP/pLL coacervate droplets are present, then an outer GOx/DEAE-dextran liquid phase gradually forms, grows around them, and eventually dissolves to produce transient multi-phase coacervate droplets. Movie is shown at ×100 real-time speed at 10 frames per seconds. Total time in real-time speed was ~ 57 minutes. Scale bar, 20 µm.

5. Supplementary references

1 C. R. Søndergaard, M. H. M. Olsson, M. Rostkowski and J. H. Jensen, *J. Chem. Theory Comput.*, 2011, **7**, 2284–2295.

2 H. Li, A. D. Robertson and J. H. Jensen, *Proteins*, 2005, **61**, 704–721.

3 N. A. Baker, D. Sept, S. Joseph, M. J. Holst and J. A. McCammon, *Proc. Natl. Acad. Sci.*, 2001, **98**, 10037–10041.

4 T. J. Dolinsky, P. Czodrowski, H. Li, J. E. Nielsen, J. H. Jensen, G. Klebe and N. A. Baker, *Nucleic Acids Res.*, 2007, **35**, W522-5.
